# An adult co-presented with varicella and herpes zoster caused by varicella zoster virus genotype J, China: a case report

**DOI:** 10.1186/s12879-020-05192-3

**Published:** 2020-06-29

**Authors:** Guangcheng Xie, Qiongling Wei, Wenping Guo, Dan Li, Pingping Sun, Jiangli Wang, Houguang Liu

**Affiliations:** 1 Department of Pathogenic Biology, Chengde Medical University, Chengde, 067000 China; 2 Department of Dermatology, The Third Hospital of Xiamen, Xiamen, 316000 China; 3 Department of Microbiology Laboratory, Chengde Center for Disease Control and Prevention, Chengde, 067000 China

**Keywords:** Varicella, Herpes zoster, Varicella zoster virus, Adult, Case report

## Abstract

**Background:**

Varicella zoster virus (VZV) causes varicella primarily in childhood, and some rare adults also report varicella. Herpes zoster mainly occurs in adults by endogenous reactivation of latent VZV. Until now, varicella and herpes zoster have seldom been reported simultaneously in one patient. Here, we report a rare case co-presenting with varicella and herpes zoster in a Chinese adult.

**Case presentation:**

A 44-year-old Chinese man suffered papules and vesicles with pain on the left ear. Five days after onset, he was admitted to the Department of Dermatology of The Third Hospital of Xiamen. Physical examination revealed that small vesicles surrounded by erythema had developed on his trunk, back and neck, and unilateral papules and vesicles in ribbons had also developed on the left ear. This patient was excluded from human immunodeficiency virus and *Treponema pallidum* infections by ELISA antibody tests. Laboratory tests revealed that the ratio of eosinophils (0.1%) and eosinophil count (0.0 × 10^9^/L) were significantly downregulated. Treatment with valacyclovir, ebastine, mecobalamine, pregabalin and calamine lotion for 5 days was effective therapy for varicella and herpes zoster. Polymerase chain reaction for vesicular fluids from varicella and herpes zoster was positive for VZV, and further phylogenetic analysis and single nucleotide polymorphism variations confirmed that the VZV genotype was type J (clade 2).

**Conclusions:**

This rare case highlights awareness of varicella and herpes zoster caused by VZV infection in adults. Our report provides novel insight into the rare clinical presentation of VZV genotype J.

## Background

Infection with varicella zoster virus (VZV) can cause trivial mucocutaneous lesions to life-threatening neurological syndrome complications, such as postherpetic neuralgia (PHN) and vasculitis [1]. Varicella (chickenpox) and herpes zoster (shingles) are two distinct clinical presentations of mucocutaneous lesions caused by VZV. Primary infection with VZV causes varicella in childhood; then, VZV establishes a latent state in peripheral ganglia, and reactivation of latently persistent virus causes herpes zoster decades later [2]. However, varicella in adult populations have been reported in India [3] and Japan [4, 5]. Several studies have confirmed concurrent varicella and herpes zoster in middle-aged and old adults [6–9], but the pathogen and the genotyping of the causative agent were not determined.

Single nucleotide polymorphisms (SNPs) in different open reading frames (ORFs) of the VZV genome have been used for VZV genotyping. Genogroups E (European), M (Mosaic) and J (Japanese) of VZV are further divided into seven clades (clade 1, E1; clade 2, J; clade 3, E2; clade 4, M2; clade 5, M1; clade 6, M4; and clade 7, M3) based on specific SNPs in ORFs 1, 21, 22, 50 and 54 [10–13]. Here, we report a rare case of varicella on the neck, trunk and back and herpes zoster on the left ear in a Chinese adult caused by VZV genotype J (clade 2).

## Case presentation

**Fig. 1:**
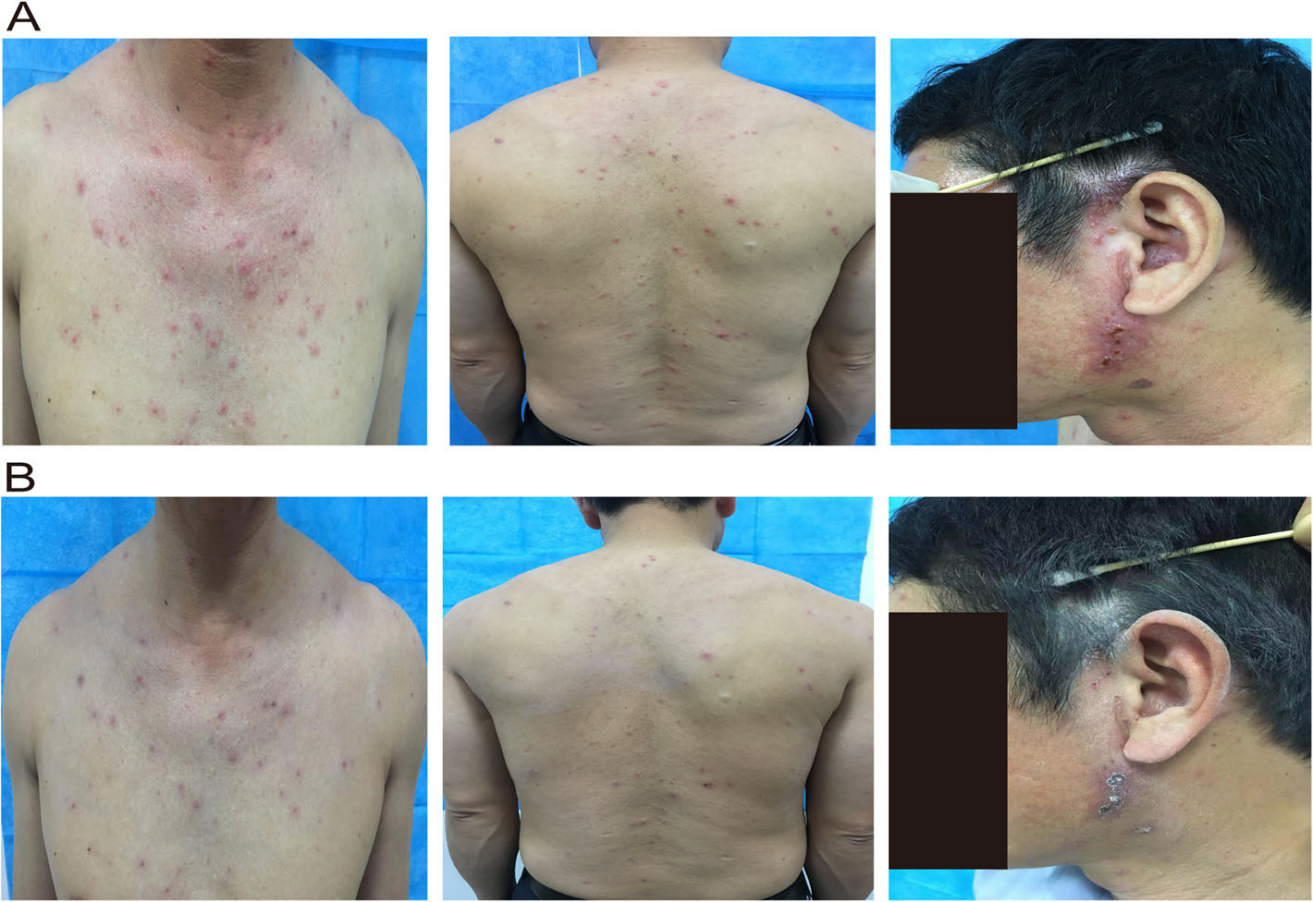
**a** and **b** Clinical presentation of varicella and herpes zoster in the patient’s trunk, back and head before or after treatment

Here, we report a 44-year-old Chinese man who was suffering from vesicles surrounded by erythema on the left ear and was admitted to The Third Hospital of Xiamen. He complained of pain lasting for 5 days. The patient said he was previously healthy without history of varicella or known drug allergic history and denied any history of medical or medication-related immunosuppression when he was admitted to the hospital. Physical examination found small vesicles (1–2 mm diameter) surrounded by erythema had developed on his trunk and back (Fig. 1a) and on the neck (Supplementary Fig. S1A). These vesicles were diagnosed as varicella. The papules and vesicles in ribbons on the left ear involved 1 to 3 dermatomes, the vesicular eruption was unilateral, lesions appeared over the first 5 days, and they were ulcerated. These lesions were diagnosed as herpes zoster (Fig. 1a). To exclude immunocompromised conditions and other diseases, the antibody titres of human immunodeficiency virus (HIV) and *Treponema pallidum* were determined, but the patient was negative for these two pathogens. Chest computed tomography confirmed the patient did not have pneumonia. The laboratory test showed that the ratio of neutrophils (82.6%) and neutrophil count (7.8 × 10^9^/L), concentrations of haemoglobin (177 g/L) and complement C4 (0.43 g/L), and haematocrit (51.3%) were upregulated; however, the ratios of lymphocytes (14.3%) and monocytes (2.6%) were downregulated; the ratio of eosinophils (0.1%) and the eosinophil count (0.0 × 10^9^/L) were significantly downregulated (Supplementary Table S1). The patient was prescribed valacyclovir, ebastine, mecobalamine, pregabalin and calamine lotion. The vesicles became crusts and then disappeared after treatment for 1 week (Fig. 1b and Supplementary Fig. S1B).

To determine the causative pathogen, vesicular fluids from varicella and herpes zoster were collected. Herpes simplex virus type 1 (HSV-1), HSV-2 and VZV were detected by nested PCR [14], and only VZV was detected (Supplementary Fig. S2A). The viral load of VZV was assessed using real-time PCR [14], and the viral load of VZV in the head (mean of Ct = 17.35) was higher than that in the trunk (mean of Ct = 18.64) (Supplementary Fig. S2B). To confirm the accuracy of the results, HSV-1, HSV-2, VZV and cytomegalovirus (CMV) were detected again [15]. Vesicles in the trunk and head were positive for VZV (Supplementary Fig. S2C).

**Fig. 2:**
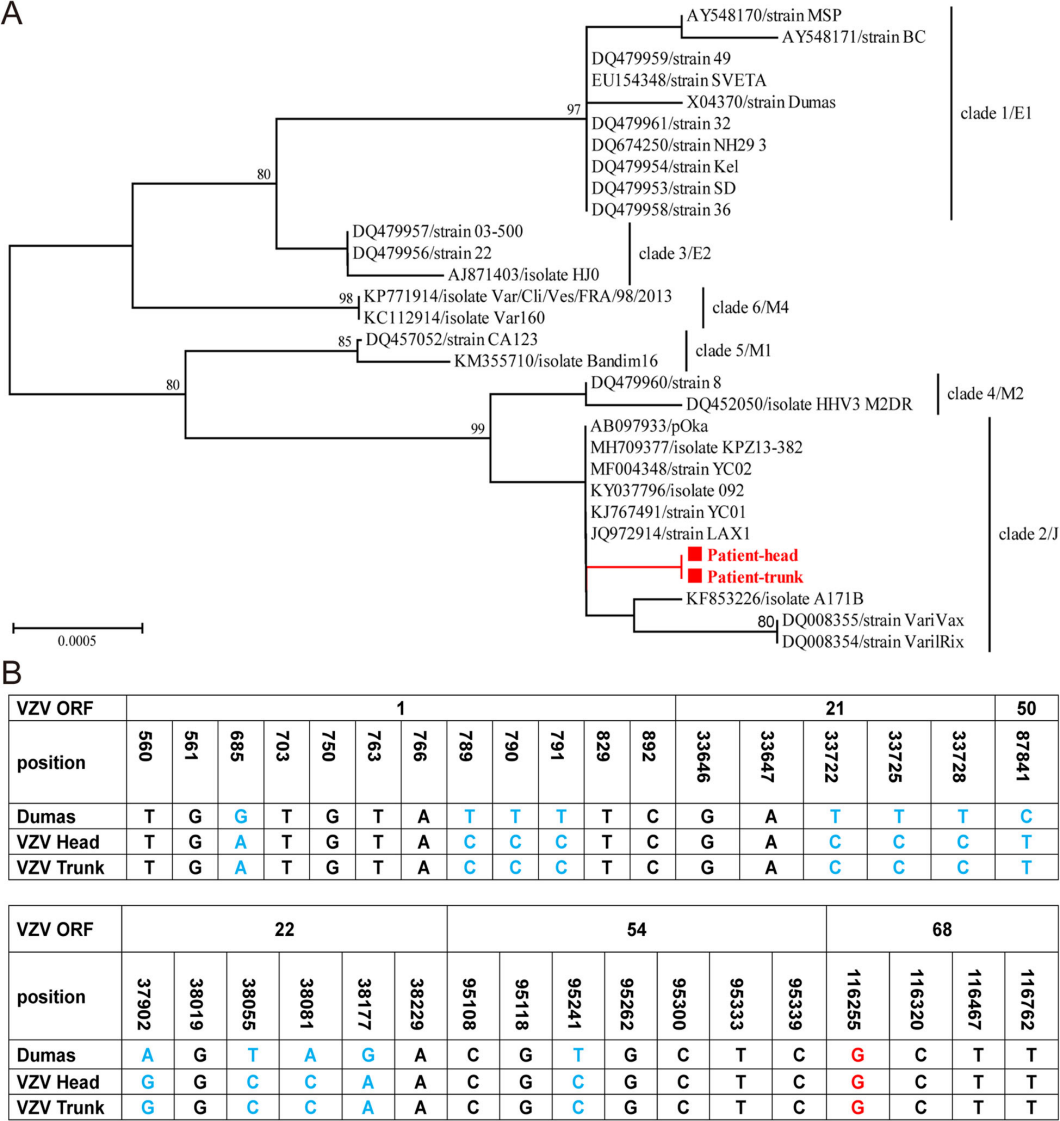
**a** Concatenated phylogenetic analysis of VZV by the neighbour-joining method with 1000 replications with the Tamura model. Values on the branches indicate bootstrap values (cutoff value 70%). Squares indicate sequences obtained in this study. Scale bar indicates nucleotide substitutions per site. **b** Identification of SNP variations of VZV based on the partial ORFs 1, 21, 22, 50, 54 and 68

To examine the genotype of VZV, 6 ORFs (ORFs 1, 21, 22, 50, 54 and 68) of the VZV genome were amplified (Supplementary Fig. S2D) [11, 13, 16], and phylogenetic analysis indicated that the genotypes of VZV in the trunk and head were genotype J (clade 2) (Fig. 2a). The SNP variations in 5 ORFs (ORFs 1, 21, 22, 50 and 54) were determined. A total of 13 SNP variations were found, and the synonymous mutations also confirmed that VZV in the trunk and head were genotype J (Fig. 2b). To assess this VZV whether a VZV glycoprotein E (gE) mutant virus (VZV-MSP), ORF68 (gE) was amplified. A synonymous G > A mutation in codon 150 of ORF68 (SNP 116255) did not occur (Fig. 2b); this result confirmed that VZV in the trunk and head was not a VZV-MSP mutant virus.

## Discussion and conclusions

Varicella epidemics in adults are not yet well studied. Previous studies have confirmed middle-aged and old adults with simultaneous occurrence of varicella and herpes zoster [6–9]; however, these rare cases with varicella and herpes zoster have seldom been reported. Here, we also described a rare case of varicella and herpes zoster in late adulthood. For this case, we excluded the possibility of contact with varicella or herpes zoster patients because his family members were healthy and he did not have a recent history of travel at home and abroad. The vesicles on the neck, trunk and back without dermatomal distribution were indistinguishable from the varicella in childhood; however, the vesicles in the left ear had dermatomal distribution and PHN, so we diagnosed this patient as a rare case. A total of 110 young university students were diagnosed with varicella during February 2016–January 2017 in India [3]. A total of 22 adult foreigners from 8 countries experienced varicella during January 2012–December 2016 in central Tokyo, Japan [5]. A 54-year-old Japanese woman also suffered from varicella caused by VZV [4]; however, this woman had secondary VZV infection because she had a high titre of VZV IgG. One of the important limitations in this study is not detecting the titre of VZV IgG because an ELISA kit was not available. The titre of HSV-2 IgM was detected, but the result was negative. Hence, we could not determine whether this patient had a secondary infection or reactivation of VZV. This rare simultaneous infection of varicella and herpes zoster in this patient was confirmed to be caused by VZV infection; however, if further differentiating between disseminated VZV infection and varicella, understanding the pathogenesis of varicella and herpes zoster occurring at the same time will provide more useful information for the treatment of VZV infection. Patients with varicella zoster disease of the central nervous system (CNS) may present without accompanying zoster rash [12], so our understanding of the molecular triggers for latent VZV remains largely unknown.

The genotypic prevalence of VZV in China showed that VZV genotype J was the major prevalent strain from 2008 to 2012. In addition, VZV genotypes M1 and M2 also circulated in Guangdong [17]. VZV genotype J was also the dominant pathogen of VZV infection in Chinese children during August 2017–September 2017 in Suzhou, Jiangsu [18]. In this study, we determined the VZV as genotype J through phylogenetic analysis and confirmed it through SNP variations in 5 ORFs. A previous study confirmed that a rare VZV genotype E caused varicella in a Japanese woman [4]. Our study confirmed that VZV genotype J also caused varicella in adults and identified that VZV was not a VZV-MSP mutant virus. Further study needs to focus on the relationship between the clinical presentation and genogroups of VZV, which will provide a useful guide for clinical doctors diagnosing VZV infection. In conclusion, we first reported that infection with VZV genotype J causes a rare case of concurrent varicella and herpes zoster in an adult.

## Supplementary information

**Additional file 1: Supplementary Fig. S1.A** Presentation of varicella on the neck, back and trunk before treatment. **B** Presentation of varicella on the neck, back and trunk after treatment.

**Additional file 2: Supplementary Fig. S2. A** Detection of HSV-1, HSV-2 and VZV by nested PCR. **B** Detection of VZV by real-time PCR. **C** Detection of HSV-1, HSV-2, VZV and CMV by nested PCR. 1 and 2 indicate vesicular fluids from the head and trunk, respectively. **D** Amplification of the 6 ORFs (ORFs 1, 21, 22, 50, 54 and 68) of the VZV genome by PCR

**Additional file 3: Supplementary Table S1.** Clinical characteristics of a rare case with varicella and herpes zoster in Xiamen, China

## Data Availability

All relevant data to this case are reported in the manuscript.

## Abbreviations

VZV: Varicella zoster virus
PHN: Postherpetic neuralgia
SNPs: Single nucleotide polymorphisms
ORFs: Open reading frames
HIV: Human immunodeficiency virus
HSV: Herpes simplex virus
CMV: Cytomegalovirus
