# Supplementary figures and images for: An adult co-presented with varicella and herpes zoster caused by varicella zoster virus genotype J, China: a case report
Source: BMC Infect Dis. 2020 Jun 29;20:454. doi: 10.1186/s12879-020-05192-3 (PMC7325039; doi:10.1186/s12879-020-05192-3)

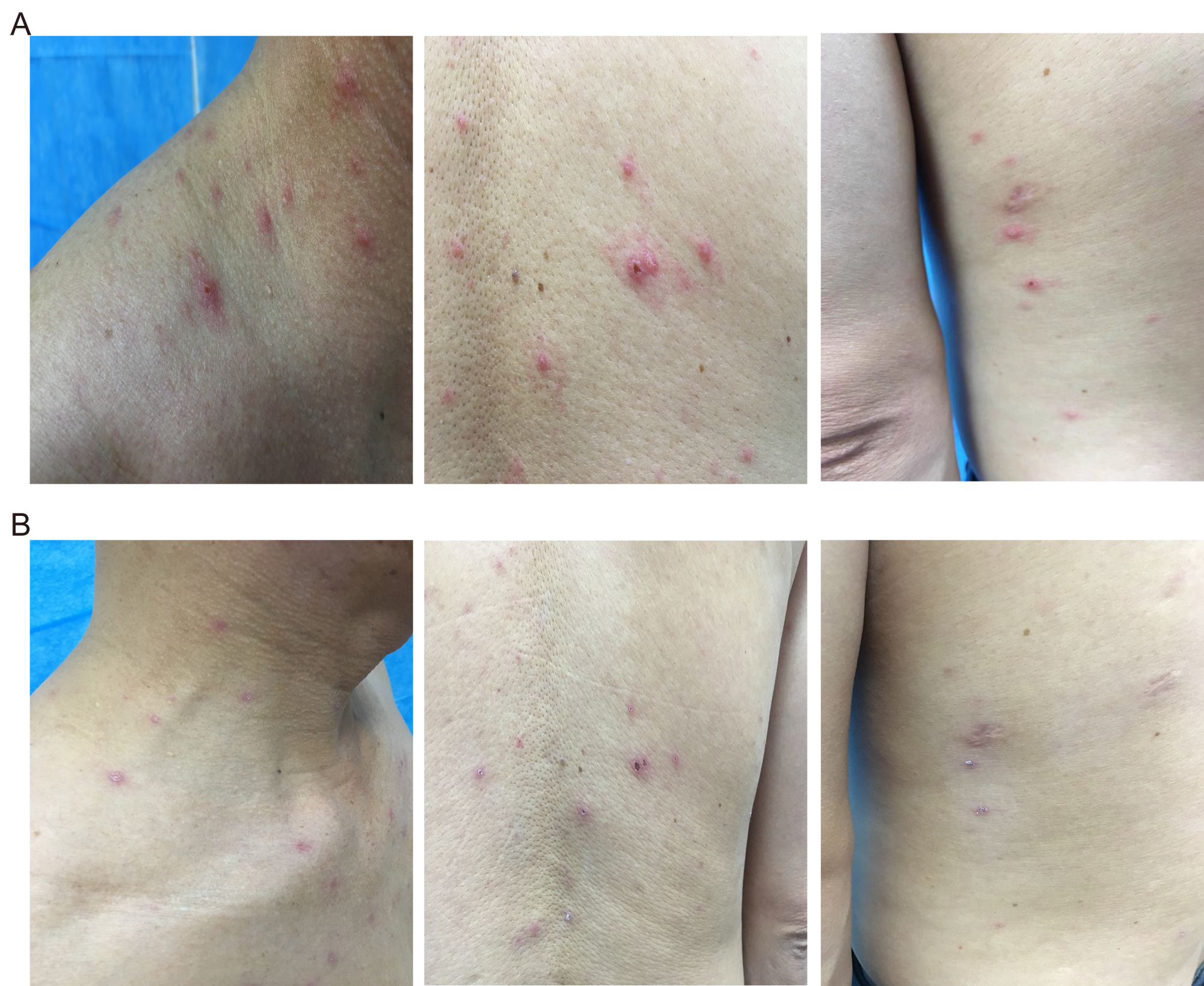

Supplement: Supplementary file 1 — Additional file 1: Supplementary Fig. S1.A Presentation of varicella on the neck, back and trunk before treatment. B Presentation of varicella on the neck, back and trunk after treatment. [file 12879_2020_5192_MOESM1_ESM.tif]

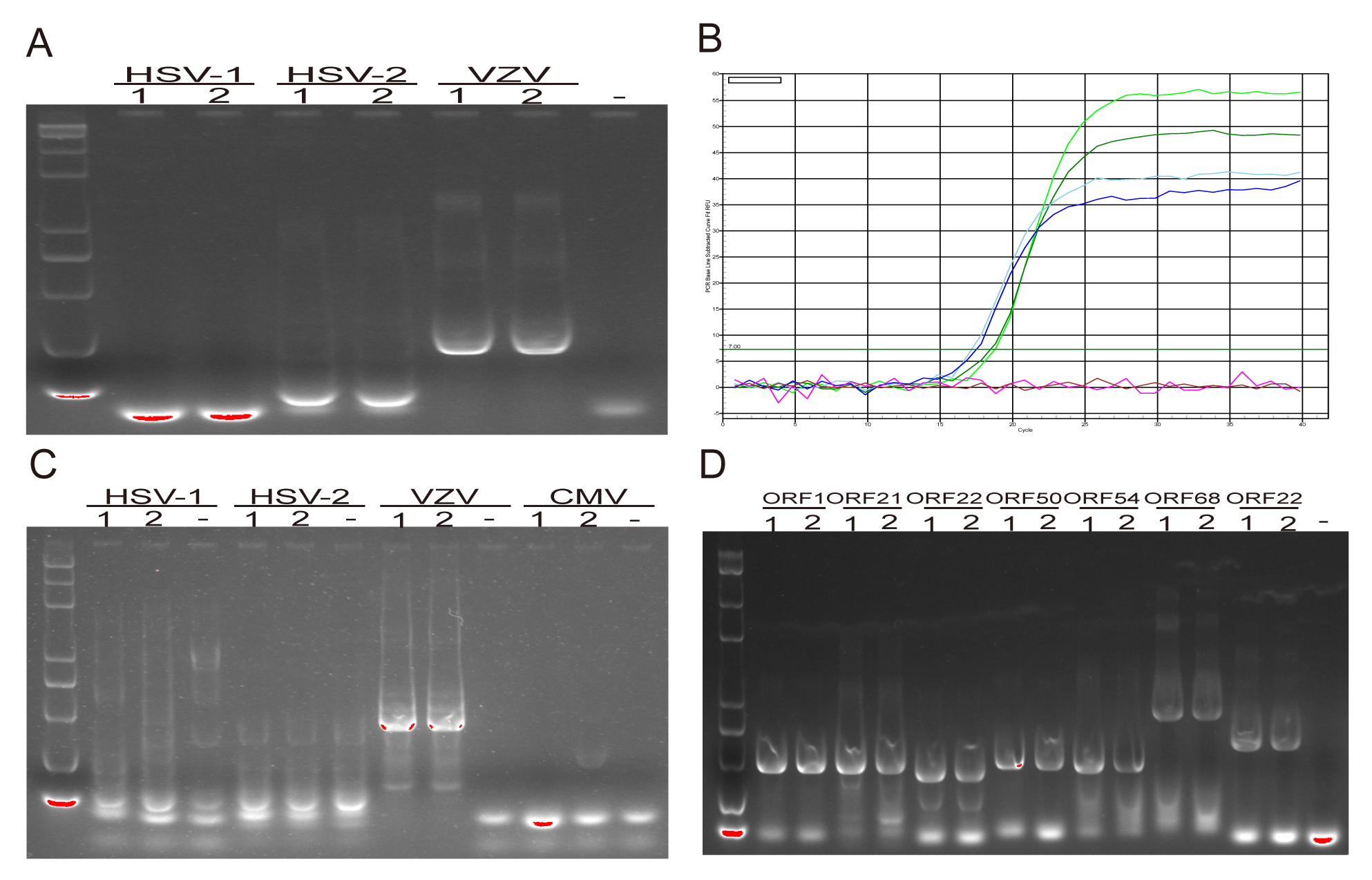

Supplement: Supplementary file 2 — Additional file 2: Supplementary Fig. S2. A Detection of HSV-1, HSV-2 and VZV by nested PCR. B Detection of VZV by real-time PCR. C Detection of HSV-1, HSV-2, VZV and CMV by nested PCR. 1 and 2 indicate vesicular fluids from the head and trunk, respectively. D Amplification of the 6 ORFs (ORFs 1, 21, 22, 50, 54 and 68) of the VZV genome by PCR [file 12879_2020_5192_MOESM2_ESM.tif]
