# Supplementary material for: An adult co-presented with varicella and herpes zoster caused by varicella zoster virus genotype J, China: a case report
Source: BMC Infect Dis. 2020 Jun 29;20:454. doi: 10.1186/s12879-020-05192-3 (PMC7325039; doi:10.1186/s12879-020-05192-3)
Supplement: Supplementary file 3 — Additional file 3: Supplementary Table S1. Clinical characteristics of a rare case with varicella and herpes zoster in Xiamen, China [file 12879_2020_5192_MOESM3_ESM.docx]

**Supplementary Table S1** Clinical characteristics of a rare case with varicella and herpes zoster in Xiamen, China

| Test categories | Patient | | Reference |
| --- | --- | --- | --- |
| WBC (×10^9^/L) | 9.5 |  | 3.5-9.5 |
| Neutrophils (%) | 82.6 | ↑ | 40-75 |
| Lymphocytes (%) | 14.3 | ↓ | 20-50 |
| Monocytes (%) | 2.6 | ↓ | 3-10 |
| Eosinophils (%) | 0.1 | ↓ | 0.4-8.0 |
| Neutrophils count (×10^9^/L) | 7.8 | ↑ | 1.8-6.3 |
| Eosinophils count (×10^9^/L) | 0.0 | ↓ | 0.02-0.52 |
| Hemoglobin (g/L) | 177 | ↑ | 130-175 |
| Hematocrit (%) | 51.3 | ↑ | 40-50 |
| CRP (mg/L) | 2.41 |  | 0.4-5.2 |
| Complement C3 (g/L) | 1.39 |  | 0.79-1.52 |
| Complement C4 (g/L) | 0.43 | ↑ | 0.16-0.38 |
